# The SADI Personal Health Lens: A Web Browser-Based System for Identifying Personally Relevant Drug Interactions

**DOI:** 10.2196/resprot.2315

**Published:** 2013-04-05

**Authors:** Ben Vandervalk, E Luke McCarthy, José Cruz-Toledo, Artjom Klein, Christopher J O Baker, Michel Dumontier, Mark D Wilkinson

**Affiliations:** ^1^James Hogg Research CentreHeart & Lung InstituteUniversity of British ColumbiaVancouver, BCCanada; ^2^Department of BiologyCarleton UniversityOttawa, ONCanada; ^3^Department of Computer Science and Applied StatisticsUniversity of New BrunswickSaint John, NBCanada; ^4^Centro de Biotecnología y Genómica de PlantasUniversidad Politécnica de MadridPozuelo de Alarcón (Madrid)Spain

**Keywords:** drug interactions, telemedicine, Web-based services, Web-based interaction, semantic Web, SADI, SHARE

## Abstract

**Background:**

The Web provides widespread access to vast quantities of health-related information that can improve quality-of-life through better understanding of personal symptoms, medical conditions, and available treatments. Unfortunately, identifying a credible and personally relevant subset of information can be a time-consuming and challenging task for users without a medical background.

**Objective:**

The objective of the Personal Health Lens system is to aid users when reading health-related webpages by providing warnings about personally relevant drug interactions. More broadly, we wish to present a prototype for a novel, generalizable approach to facilitating interactions between a patient, their practitioner(s), and the Web.

**Methods:**

We utilized a distributed, Semantic Web-based architecture for recognizing personally dangerous drugs consisting of: (1) a private, local triple store of personal health information, (2) Semantic Web services, following the Semantic Automated Discovery and Integration (SADI) design pattern, for text mining and identifying substance interactions, (3) a bookmarklet to trigger analysis of a webpage and annotate it with personalized warnings, and (4) a semantic query that acts as an abstract template of the analytical workflow to be enacted by the system.

**Results:**

A prototype implementation of the system is provided in the form of a Java standalone executable JAR file. The JAR file bundles all components of the system: the personal health database, locally-running versions of the SADI services, and a javascript bookmarklet that triggers analysis of a webpage. In addition, the demonstration includes a hypothetical personal health profile, allowing the system to be used immediately without configuration. Usage instructions are provided.

**Conclusions:**

The main strength of the Personal Health Lens system is its ability to organize medical information and to present it to the user in a personalized and contextually relevant manner. While this prototype was limited to a single knowledge domain (drug/drug interactions), the proposed architecture is generalizable, and could act as the foundation for much richer personalized-health-Web clients, while importantly providing a novel and personalizable mechanism for clinical experts to inject their expertise into the browsing experience of their patients in the form of customized semantic queries and ontologies.

## Introduction

For the lay-person, free health information on the Web is a potentially valuable resource for better understanding personal symptoms, medical conditions, drugs, and nutrition. However, while sites such as WebMD [1] provide accredited sources of information, a multitude of other sources such as blogs, Wikipedia articles, and news articles exist that provide no guarantees about their completeness, accuracy, or objectivity. Thus, distinguishing between reliable and unreliable information is a central issue. However, even when restricted to verified sources, the sheer quantity of available material often makes meaningful interpretation a challenging task for readers without a medical background. For example, a study published by White and Horvitz in 2009 demonstrated a phenomenon known as “cyberchondria”, wherein a person experiences a heightened anxiety about medical conditions after reviewing search results and online literature relating to common symptoms (eg, headache [2]). Finally, the issue of “information quality” is both personal and subjective—information that is relevant for one individual might not be relevant to another, based on personal factors such as overall health, drug-regimen or other interventions, lifestyle, gender, or even personal interest. As observed by Burgess et al, “due to the unique nature of every individual, a single…method that assumes all people are the same will fail to meet every person’s information needs” [3]. It would be highly desirable, therefore, to assist Web users by filtering and/or annotating the information they encounter on the Web in a personalized way, thereby increasing its relevance and utility to that individual. Moreover, given that a patient may be at any point in their continuum-of-care, it would be particularly desirable to provide the patient’s clinical-care team an opportunity to include their “voice” into that annotation and filtering mechanism, such that the clinician’s plan for that patient enhances the personalization of the page even further.

We recently published a novel framework for automatically synthesizing contextually-sensitive data retrieval and analysis workflows composed of dynamically-selected Semantic Web Services [4]. The system used a semantic template describing the abstract “intent” of the workflow, combined with the specific details in a given dataset, to concretize an appropriate workflow capable of evaluating that data. In this work we intend to demonstrate, using a straightforward prototype application called the Semantic Automated Discovery and Integration (SADI) Personal Health Lens, that these components—the data, the semantic model, and the derived analytical workflow—map directly onto the various facets of the Health Web personalization problem described above. The Personal Health Lens application utilizes the patient’s individual medical history and data, the clinician’s plan for that patient (in the form of a semantic query), and publicly available tools that can be automatically selected and appropriately chained-together to analyze and contextually interpret the content of a webpage. The output is an annotated page containing new information specifically relevant to that patient, which is loaded into their browser as usual.

Personalization of Web information necessitates the use of the individual’s personal medical profile, since this information can be used to identify both relevant and inappropriate advice within a webpage. There has been considerable work in the area of semantic representation of electronic patient records, both institutional [5] and personal [6], and a variety of different standards exist for the content and structure of these records. Here, we do not intend to prescribe or proscribe any particular standard. This work simply assumes that such health records exist, are accessible to the local browser, and are (or can be) expressed in standard Semantic Web syntaxes. As the various models for electronic health records begin to consolidate, the prototype system could be easily re-written to conform to that standard. For the purposes of this prototype, we created a simple model of the drug-regime portion of the patient’s health record, along with several other clinical and personal features.

Much of the prior work in “personalization” of a patient’s Web experience has been in the domain of facilitated search, particularly through the use of semantically-backed personal health portals (eg, [3,7]) where the role of semantics in the portal is either for search-expansion to discover additional relevant pages, and/or to simplify medical terminology for the patient while still providing access to complex literature. While portals allow a much higher degree of potential curation by experts, thus (presumably) improving information quality, they also require a much higher level of buy-in from the patient, who must choose to exclusively visit the portal for their health information in lieu of more typical forms of Web exploration such as search engines and browsing. Moreover, though a portal may provide access to a curated or filtered set of pages, the pages themselves are generally untouched; we were unable to identify any personal health portal in which the source pages were, themselves, contextually marked-up. We believe that context is an important part of the personalization experience, as is the freedom to explore the Web in any manner the patient chooses. The Personal Health Lens, therefore, was designed to operate over any page, at any time, discovered in any manner, and places its patient-oriented annotations directly into the page-context. This is achieved by utilizing the semantics—both in the patient’s data, as well as in a semantic template query—to generate a contextually-relevant Semantic Web Service-based annotation workflow at the moment the Health Lens is invoked. This workflow may differ depending on the patient’s medical information and the content of the page they are viewing, thus providing the flexibility necessary to operate on the open Web, rather than through a curated portal.

We now describe in detail our prototype system that attempts to identify drugs within the online literature that are potentially dangerous to the user based on their personal health record. We begin by giving an overview of the system and a description of the prototype implementation in the Methods section. In the Results section, we demonstrate use of the prototype in the context of a sample health profile. Finally, we discuss some high-level design issues and provide closing remarks in the Discussion section.

## Methods

### Use of Semantic Web Standards and Technologies

#### RDF, OWL, SADI, and SHARE

##### Overview

All data within the Health Lens system were represented using the Resource Description Framework (RDF) model [8], which provides significant advantages over other formats such as Extensible Markup Language XML and ad hoc text formats when integrating data across multiple sources. In particular, RDF allows the assignment of globally unique identifiers (URIs) to all entities within the data, making it possible to match entities across distributed data sets in a reliable manner. Further, RDF supports an automated procedure called RDF-Merge [9] for merging data sets into a single file or database, regardless of the actual content of the data sets involved. This significantly reduced the cost of developing a database for the system, and will also reduce the cost of maintenance when adding new data sources in the future. Finally, in contrast to other data models, RDF data sets are not constrained to a fixed schema. This property of RDF allows any type of statement to be added to an existing data set without adversely affecting clients or servers that use that data set. For instance, in Health Lens, the use of RDF to represent the personal health profile will allow any new type of health data to be added to the profile in the future without requiring an update to the database schema or the interfaces of the various system components.

The Web Ontology Language (OWL) [10] also played a central role in the design of the Health Lens system, providing a general framework for describing and processing logical constraints about RDF data. The chief advantage of using OWL in the context of the Health Lens project is that data matchmaking and validation tasks can be performed by a reusable tool called an OWL reasoner (eg, [11]), rather than by custom, application-specific software.

The Health Lens system utilizes 2 Semantic Web projects that have been built on top of the RDF and OWL standards—Semantic Automated Discovery and Integration, and Semantic Health and Research Environment.

##### Semantic Automated Discovery and Integration (SADI)

SADI is a set of design patterns for producing stateless Web services that natively consume and produce RDF data. The structure of the input and output data for SADI services are formally described by an input OWL class and an output OWL class, respectively. Further details about SADI are described in [12].

##### Semantic Health and Research Environment (SHARE)

SHARE is a proof-of-concept Semantic Web query engine that resolves SPARQL Protocol and RDF Query Language (SPARQL, [13]) queries by building and executing workflows of SADI services. SHARE is also capable of discovering instances of a given OWL class by building an appropriate SADI workflow. Further details about SHARE are described in [14].

#### A Note About Opaque URIs in RDF Data

Many of the URIs in the example RDF data of this paper are opaque, making human interpretation of the data considerably more difficult than if human-readable identifiers had been used. For example, the URI of the “has attribute” predicate of the Semanticscience Integrated Ontology (SIO) ontology [15] is http://semanticscience.org/resource/SIO_000008. The use of opaque URIs is a best practice when the uniqueness of a label for an entity cannot be guaranteed, due to the existence of synonymous labels or labels in multiple languages. By convention, human-readable labels are indicated via the *rdfs:label* predicate. This allows multiple labels to be assigned to the same entity, and also permits changes to the labels without adversely affecting other RDF data sets on the Web that refer to the entity in question. To aid the reader, comments are provided within the example data that provide translations from opaque URIs to human-readable labels.

### System Overview

**Figure 1:**
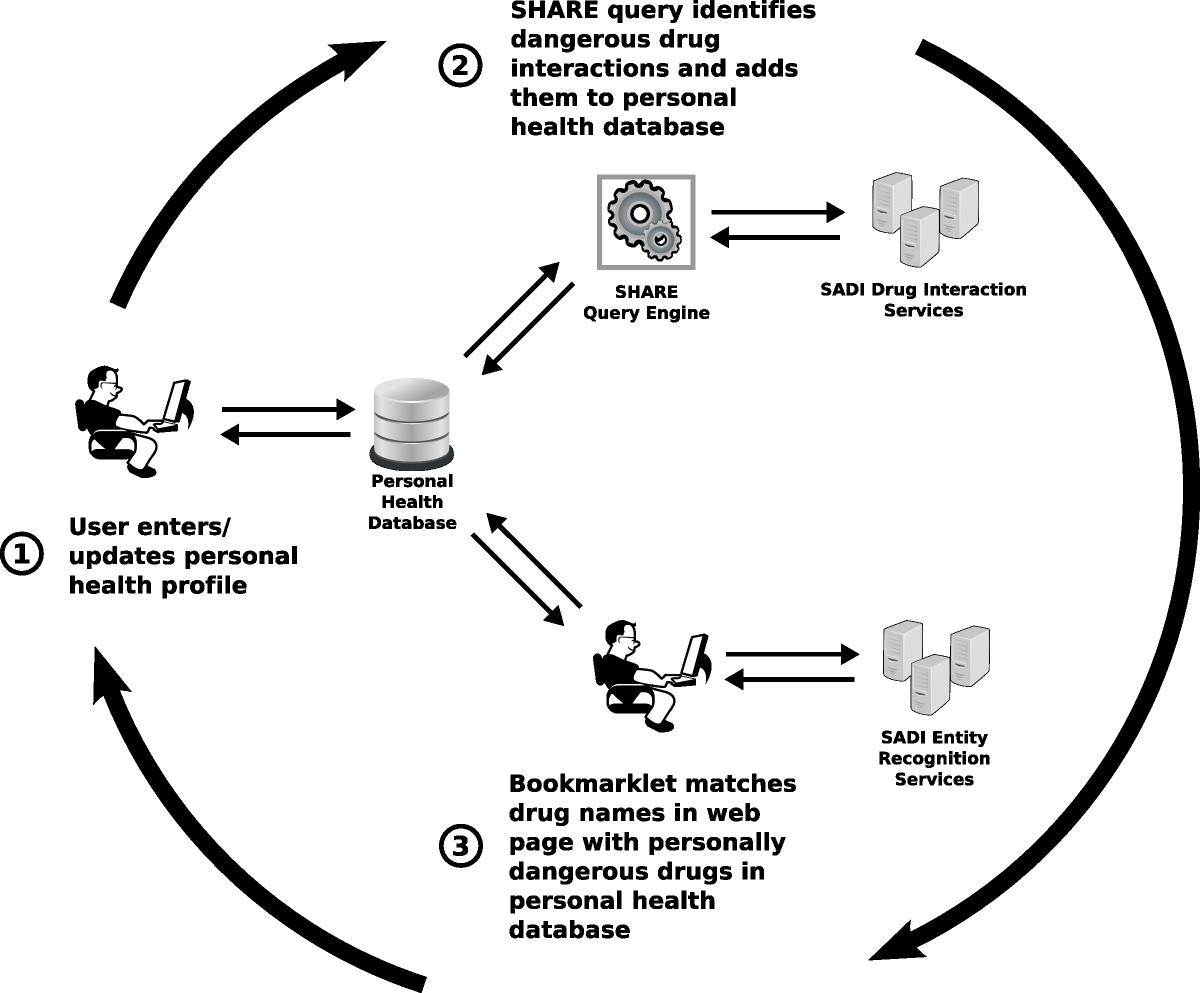
An overview of the SADI Personal Health Lens system. In Step 1, the user enters or updates his/her personal health profile, which contains information such as sex, age, and current prescriptions. In Step 2, a SHARE query is used to aggregate interactions of any known drug with the user’s personal health profile. This query may be time-intensive and is performed as a separate step prior to interactive use of the system. In Step 3, the user browses the Web and clicks the Health Lens bookmarklet when reading a health-related webpage. The bookmarklet identifies drugs mentioned within the webpage using one or more SADI services for entity recognition, and compares those drugs with drugs in the personal health database that are known to be personally dangerous. Finally, the bookmarklet annotates the webpage with appropriate warnings.

The Health Lens system operates in a cycle of 3 steps, as depicted in Figure 1:

1. The user creates or updates his/her *personal health profile*, which is stored locally in the *personal health database*.
2. The system executes a SHARE query to identify dangerous interactions between the user's personal health profile and any known drug. All discovered interactions are aggregated and stored in the personal health database for use in Step 3.
3. From within a Web browser, the user clicks the Health Lens bookmarklet to identify mentions of drugs in the current webpage that may be personally dangerous. The bookmarklet accomplishes this by (a) identifying drug mentions within the text using one or more SADI entity recognition services, and (b) comparing the identified drugs against the list of drugs that have interactions with the personal health profile, as computed in Step 2.

The circular arrows of Figure 1 indicate that Steps 1-3 occur in a repeating cycle; each time the user changes his/her profile (eg, adding a new prescription), Step 2 must be rerun to rebuild the full set of known interactions with the profile.

### Step 1: Creating/Updating the Personal Health Profile

Our prototype implementation of the Health Lens system includes a sample personal health profile (Multimedia Appendix 1). The profile is provided as an RDF/N3 file, and describes a 37-year-old female named Jane Doe who is on a prescription for Alprazolam (Xanax), a member of the benzodiazepine class of psychoactive drugs that is commonly used to treat anxiety disorders.

While the current focus of the profile is drug prescriptions, the use of RDF will allow future extension of the profile to include personal data such as allergies, medical conditions, diet, clinical test results, and genotyping.

Currently, changes to the health profile must be made by editing the RDF/N3 file by hand; however, any future versions of the system will provide a user interface for this task. The schema for the sample health profile uses a combination of Friend of a Friend (FOAF) [16], an ontology for describing people and their relationships, and the SIO [15], an upper level ontology for representing scientific data. The main subject of the profile, Jane Doe, is represented by a blank node (“[ ]”) with an *rdf:type* of *foaf:Person*, and this node has a combination of FOAF and DBPedia [17] properties indicating first name, last name, gender, and date of birth. A prescription node is attached to the Jane Doe node by the *med:prescription* property, and this node has attached attributes indicating the prescribed drug, quantity, dosage, and date of prescription.

### Step 2: Precomputing Dangerous Drug Interactions

In order to access dangerous drug interactions, we developed a drug-drug interaction (DDI) discovery SADI service. For the backend data, a small data set of approximately 40 known drug interactions with St. John’s Wort, a herbal treatment for depression, was constructed manually from the literature. The authors chose to use a hand-constructed data set for the initial version of the project because they were unsuccessful in finding a curated public resource with all of the required details of the interactions, namely directionality, supporting citations, and coded clinical effects. The databases that came closest to meeting the requirements were DrugBank [18], which describes clinical effects in natural language, the Twosides [19] PharmGKB database, which provides only computationally-predicted interactions, NDF-RT [20], which does not provide access to supporting citations, and the Drug Interaction Knowledge Base (DIKB) [21], which does not describe clinical effects. In future work, the authors may be able to replace the hand-curated St. John’s Wort data set with some combination of these resources.

Example input and output data from the DDI service are provided in Multimedia Appendix 2 and Multimedia Appendix 3, respectively. Using the terminology of this example input/output data, the DDI discovery service takes as input a “chemical entity” (Alprazolam) that “has attribute” some “chemical identifier” and outputs an “annotated chemical entity” that “is participant in” some “drug-drug interaction”. The provided output data describes a directed drug-drug interaction between St. John’s Wort and the input drug Alprazolam, where the direction of the interaction is indicated by assigning the roles of “actor” and “target” to St. John's Wort and Alprazolam, respectively. A citation providing evidence for the interaction is attached via the SIO “has source” property, and the clinical effect of the interaction is attached via the SIO “results in” property. The effect of the interaction itself is represented using a controlled vocabulary term for “decreased efficacy of drug”. For clarity of exposition, many long auto-generated URIs (UUIDs) in the real service output data have been replaced by blank nodes in Multimedia Appendix 3, however, the resulting RDF is semantically equivalent.

The Health Lens system invokes the drug interaction service via a SPARQL query (Multimedia Appendix 4) to a local instance of SHARE. The result of this query is the collection of all known interactions between the user’s prescribed drugs and any other drug. Using the SHARE system as the client for the drug interaction service had several advantages over implementing application-specific client code. One advantage is that new sources for interaction data can be added to the system in the future simply by adding entries to SHARE’s registry of SADI services. Another advantage is that the nature of the conflicts that are discovered by the system can be changed by modifying the SHARE query, rather than modifying the application code. For example, while the current version of the system searches for interactions with prescribed drugs, future versions might also take into account interactions with allergies or specific genotypes.

One caveat of using SHARE system for invoking the DDI service is that running the query can be time intensive. Resolving a SHARE query involves several potentially expensive operations such as downloading ontologies, performing OWL reasoning to match local data to available services, and invoking SADI services over the Web. The Health Lens system therefore runs the SHARE query “offline”; whenever the user creates or updates his/her personal health profile, the query is re-executed and the retrieved interactions are cached in the personal health database. This approach enables the user to scan a webpage and view any resulting warnings in a reasonably short amount of time*.*

### Step 3: Identifying Dangerous Drugs in Webpages

The Health Lens system was integrated with the user's Web browser via a javascript bookmarklet [22]. Whenever the user is reading a health-related webpage, he/she can click the bookmarklet to scan the page for drugs that are potentially dangerous with respect to his/her health profile. The bookmarklet performs the following actions when activated. First, the text of the *<body>* element of the webpage is extracted using jQuery’s *text()* function. This text is then substituted into an input RDF template for the entity recognition service (described below) and the service is invoked with an AJAX request. When the service invocation completes, the URIs of the identified drugs are extracted from the output RDF. Next, the URIs of all personally dangerous drugs are obtained by issuing a SPARQL query (Multimedia Appendix 5) against the local personal health database. Finally, the URIs of all personally dangerous drugs are compared with the URIs of drugs returned by the entity recognition service, and any drugs in both of these sets are marked up with appropriate warnings in the page.

As part of the Health Lens prototype, a SADI entity recognition service was created that identifies drug names within plain text. Example input and output RDF data for the service are provided in Multimedia Appendices 6 and 7, respectively. The input data for the service was modeled using a combination of the Bibliographic Ontotology (BIBO) [23] and the Dublin Core (DC) [24] ontology, while the output data was modeled using the FOAF and SIO ontologies. A valid input is an instance of the *bibo:Document* class, with the text content attached via the *bibo:content* predicate and a MIME type of “text/plain” attached via the *dc:format* predicate. The resulting output attaches instances of SIO “chemical entity” to the input document URI via the *foaf:topic* predicate, where each “chemical entity” has attributes indicating the DrugBank [18] identifier and any synonymous drug names. The semantics of *foaf:topic* are slightly stronger than is required in the context of the entity recognition task; however, since we failed to find a more suitable predicate, we followed a weaker interpretation where any object mentioned in the text was considered a topic of the document.

For the business logic of the entity recognition service, we implemented our own drug extraction algorithm based on a dictionary lookup. The dictionary was extracted from the DrugBank database and contains 58,708 entries, where each entry consists of the name of a drug, its DrugBank ID, and its canonical name in DrugBank. Drugs are recognized using the GATE ANNIE Gazetteer lookup tool [25], which loads the dictionary and annotates the text with DrugBank IDs and canonical names. At the final step, annotations of names with length less than 4 characters were filtered from results. The entity recognition service is a prototype and has not yet been evaluated in terms of precision and recall. Although it follows a basic lookup-based approach with naive filtering of candidates, it has shown qualitatively acceptable performance during testing of the Health Lens system.

## Results

The authors have provided a downloadable demonstration of the Health Lens system as a standalone executable jar file (Multimedia Appendix 8). This jar file bundles all components of the prototype system, including the sample personal health profile, the SHARE query engine, the bookmarklet, and the SADI services for drug interactions and entity recognition. Please note that running the prototype requires a Java Runtime Environment (JRE) to be installed on your system.

To start the system the user must execute the jar file, which can be done on most systems by double-clicking the file. Alternatively, the user may run the jar by typing the following at a Linux/Windows/OSX command line prompt: *java -Xmx1g -jar personal-health-lens.jar*. Executing the jar file brings up a console that allows the user to start and stop the system on port 8080. (Please note that the current version of the system will only work correctly on port 8080.) Clicking "Start" starts up the local SADI services and the Joseki server that hosts the personal health database.

In most environments, the *Start* button will open the installation/testing page for the Health Lens bookmarklet in the user's default Web browser. If this does not happen automatically, the user must instead manually visit *http://localhost:8080/* in a Web browser. The Health Lens bookmarklet can then be installed by dragging one of the two "Personal Health Lens" links near the top of the page onto the browser's bookmarks toolbar.

**Figure 2:**
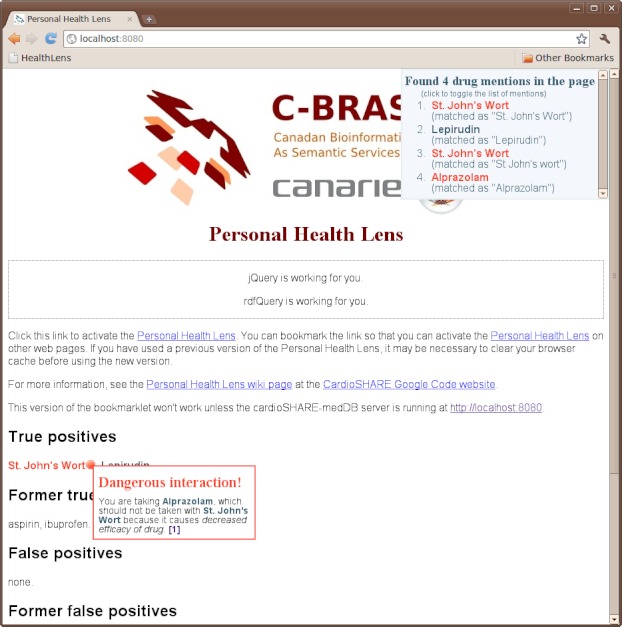
The test page for the Health Lens system, after being scanned with the bookmarklet. St. John’s Wort is annotated with a warning due to a conflict with Alprazolam, a prescribed drug in the sample personal health profile. The test page provides a mechanism to verify that all components of the system are working correctly, independently of the varying structure and content of real webpages.

The webpage at *http://localhost:8080/* can be used to test correct operation of the system. As the content of the test page is under the control of the authors, it provides a reliable means to separate issues with the system components from issues that are specific to the content of particular webpages. To run the bookmarklet against the test page, the user must click the Personal Health Lens bookmarklet in the toolbar, which will activate scanning of the page for dangerous substances. During scanning, an animated SADI logo appears in the top right hand corner of the page. When scanning is complete, the words "St. John's Wort" are colored in red and annotated with a stop sign symbol indicating a dangerous interaction. Clicking on "St. John's Wort" displays a dialog box with the details of the interaction between "St. John's Wort" and the personal health profile, as shown in Figure 2. The warning indicates that St. John's Wort may decrease the efficacy of Alprazolam, a prescribed drug in the user's personal health profile. As evidence, it provides a link to a publication that describes the interaction, which is believed to due to the induction of the CYP3A4 enzyme.

**Figure 3:**
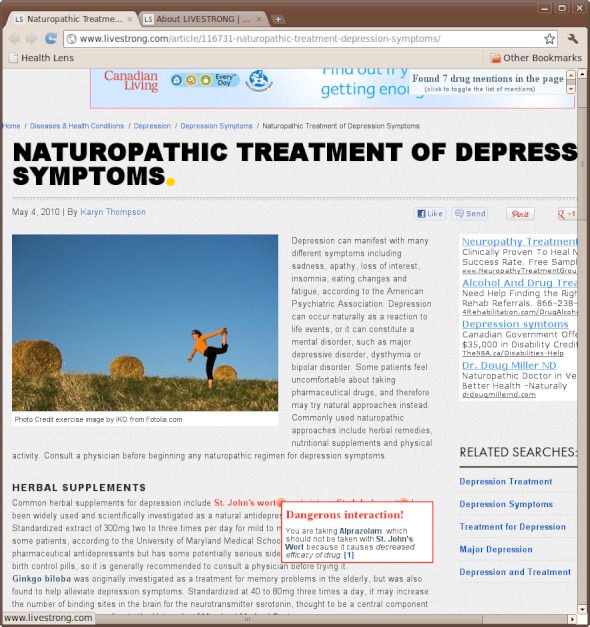
A screenshot of the Health Lens bookmarklet operating on a real webpage. The subject page correctly advises its readers to consult a physician before taking St. John’s Wort and mentions a possible interaction with birth control pills. Health Lens augments this information with a warning about an interaction between St. John’s Wort and the user’s current prescription for Alprazolam.

Any webpage may be scanned for personally dangerous drugs by clicking the Health Lens bookmarklet. For example, Figure 3 shows the St. John's Wort warnings in the context of an online article regarding naturopathic treatments for depression [26].

## Discussion

The Personal Health Lens is only one of many tools that have been developed in recent years to leverage personal health data. For example, profile-oriented smartphone applications are now available for identifying allergy-safe foods [27,28], monitoring sleep patterns [29,30], and sharing medical information across physicians and caregivers [31,32]. The use of such personalized tools is likely to continue increasing as new forms of personal health data, such as genotyping and whole genome sequencing, become more readily available to the general public. What makes the Personal Health Lens architecture distinct from other personalization tools is that, in addition to the patient’s data and the set of analytical tools, there is a third component—the semantic model/query—which guides the selection of which tool to use given which set of data. Though in this prototype the semantic model was quite simplistic, taking the form of a SPARQL query relating to drug-drug interactions, the SHARE resolution engine is capable of interpreting significantly more complex models, for example, the clinician’s treatment trajectory for any given patient. As such, we believe this is the first personal health tool which has the potential for clinician-initiated personalized medical advice to be injected into the patient’s own health research browsing activities. Moreover, since the SADI Personal Health Lens has a flexible underlying infrastructure, we believe it can be easily adapted to operate in combination with many of these existing health-information-support tools to enhance the relevance, detail, or quality of their personalization.

The Personal Health Lens project was intended only as a prototype of a novel framework for patient/practitioner/Web interaction, and was not intended to be used by genuine patients. There are some important factors of the personal profile that are not yet taken into account when detecting relevant interactions, such as sex, age, drug dosages, and drug routes (eg, pills, creams, inhalers), to name a few. Beyond these issues, it is useful to point some general concerns with our approach to health browsing personalization, and possible solutions.

Unlike earlier efforts [7], the Health Lens makes no attempt to evaluate the quality of the information on any given page; “hazardous” pages containing overtly incorrect information are not automatically weeded-out, as they are in other portal-based approaches. If there were a public registry of evaluated webpages, this limitation could be partially overcome by adding that registry’s evaluation as part of the annotation pipeline; however, it could not be guaranteed that any page the reader had accessed would exist in that registry. Thus, by ensuring that our systems scales to the Web, we become limited in our ability to filter hazardous or fallacious information. Conversely, the Health Lens system, by not adopting a portal-like structure, becomes a more natural part of the patient’s Web exploration, and one that is under their control. Rather than appearing as a centralized “paternalistic” [5] information source, the Personal Health Lens prototype allows the patient to choose when to bring the expert evaluation framework into their Web experience.

Although Health Lens does not attempt to evaluate the quality of information on webpages, information quality is still a relevant issue with respect to the underlying sources of drug interaction data. In this sense, much of the previous work and lessons learned from designing and maintaining portal-based sites such as MedlinePlus [33] could be usefully applied to Health Lens to build a more transparent and trustworthy system. For example, many metrics (“instruments”) have been developed for systematically evaluating information quality on webpages that might also be used to evaluate the quality of the drug interaction services. Existing information quality metrics are based on factors such as provision of article metadata (eg, authors, date of last update, supporting citations), accuracy of information, completeness of information, and balanced presentation of evidence [34]; there also exist various composite scoring systems, such as DISCERN [35], that employ weighted combinations of these factors. The criteria of completeness is particularly relevant to the drug interaction data sets, as there exists a potential danger that users will assume a substance is safe in the absence of any warnings about interactions. Building a perfectly comprehensive and up-to-date corpus of drug interaction data is obviously not a feasible goal, especially given the distributed nature of the system. This caveat must be made clear to the end users, and they must be reminded to consult a physician before making changes to their medication regimes, regardless of any warnings that the system does or does not report.

Another area where the system can potentially be improved is in catering to the user’s personal level of health literacy. While Health Lens makes no attempt to filter the webpages themselves by literacy level, the user’s literacy is still relevant for the appropriate presentation of warnings. Although we have intentionally described interactions in terms of high-level clinical effects (eg, “decreased efficacy of drug”), the supporting citations provided with the interactions are scientific publications and thus require a high level of medical knowledge to understand. In future work, providing references to trusted secondary sources such as MedLine Plus would probably be more appropriate for most users. As with the issues of information quality described above, our approach to accommodating the user’s literacy level could likely be informed by previous work. For instance, several ontology-based systems have been developed for automatically mapping expert medical terminology to equivalent consumer-friendly terminology (eg, [36,37]), and these might be employed within Health Lens to generate more accessible warning messages. In addition, many sociological studies have employed metrics based on word complexity and sentence length (eg, the Fry Readability Formula) to gauge readability levels of health-related webpages in a systematic manner [34]. These metrics might likewise be employed within the Health Lens framework to help present supporting documents for interaction warnings at an appropriate level of literacy.

Finally, it is important to note that there is a potential privacy concern relating to the use of a public drug interaction service—the system must send the URIs of the user's prescribed drugs out over the Internet in order to retrieve the relevant interactions. In general, there is a trade-off between the degree of privacy the user wants to maintain and the ability to utilize all possible sources of data for identifying conflicts. In future work, this issue can be addressed by allowing the user to indicate which types of data are safe to send over the Web. In addition, the user might also choose to restrict the system to services that support HTTPS. With respect to privacy, the prototype implementation errs on the side of caution by using a locally hosted drug interaction service.

We believe the SADI Personal Health Lens demonstrates that dynamically-generated, context-sensitive pipelines of data and analytical Semantic Web Services can be usefully applied to the personalization of health information on the Web. Moreover, this approach provides the unique ability to fine-tune these workflows through the utilization of expert knowledge within semantically-encoded guidelines. Thus, for the first time, the Personal Health Lens establishes a framework within which clinicians can provide on-demand guidance during their patient’s personal health explorations.

## Multimedia Appendix 1

A sample personal health profile for the Health Lens system.

## Multimedia Appendix 2

Sample input RDF for the SADI drug interaction service.

## Multimedia Appendix 3

Sample output RDF for the SADI drug interaction service.

## Multimedia Appendix 4

The SPARQL query issued to the SHARE system, in order to precompute all known drug interactions with the personal health profile.

## Multimedia Appendix 5

The SPARQL query issued by the bookmarklet, in order to obtain personally dangerous drugs from personal health database.

## Multimedia Appendix 6

Sample input RDF for the SADI entity recognition service.

## Multimedia Appendix 7

Sample output RDF for the SADI entity recognition service.

## Multimedia Appendix 8

SADI Personal Health Lens Prototype Java ARchive File [38].

## Abbreviations

AJAX: Asynchronous Javascript and XML
BIBO: Bibliographic Ontotology
DC: Dublin Core
DDI: drug-drug interaction
FOAF: Friend of a Friend
JRE: Java Runtime Environment
OWL: Web Ontology Language
RDF: Resource Description Framework
SADI: Semantic Automated Discovery and Integration
SHARE: Semantic Health and Research Environment
SIO: Semanticscience Integrated Ontology
SPARQL: SPARQL Protocol and RDF Query Language
URI: Uniform Resource Identifier
UUID: Universally Unique Identifier
XML: Extensible Markup Language
